# Impact of root exudates on soil reconstruction and bacterial community resumption in open-pit coal mines

**DOI:** 10.3389/fmicb.2025.1466452

**Published:** 2025-07-30

**Authors:** Zhuo Yang, Jianzhi Niu, Tong Wu, Jiaqi Li, Linus Zhang, Xiongwen Chen, Ronny Berndtsson

**Affiliations:** ^1^School of Soil and Water Conservation, Beijing Forestry University, Beijing, China; ^2^China Coal Technology & Engineering Group Shenyang Engineering Company, Shenyang, China; ^3^State Key Laboratory of Efficient Production of Forest Resources, Beijing, China; ^4^Key Laboratory of State Forestry Administration on Soil and Water Conservation and Desertification Combating, Beijing, China; ^5^Engineering Research Center of Forestry Ecological Engineering of Ministry of Education, Beijing Forestry University, Beijing, China; ^6^College of Land and Environment, Shenyang Agricultural University, Shenyang, China; ^7^Division of Water Resources Engineering and Centre for Advanced Middle Eastern Studies, Lund University, Lund, Sweden; ^8^Department of Biological and Environmental Sciences, Alabama A&M University, Huntsville, AL, United States

**Keywords:** root exudates, open-pit coal mines, soil reconstruction, bacteria community, diversity and abundance

## Abstract

Open-pit coal mine reconstructed ecosystems are ecologically fragile. Retained early stage topsoil is usually not enough to maintain plant growth. For this purpose, we used root exudates to fertilize the reconstructed soil and improve the functioning of the soil microorganism ecology. The roots’ exudates increased the concentration of organic matter and total nitrogen by 16–39%. Within a certain concentration range, the higher the concentration of root exudate, the higher the soil fertility. When the concentration of root exudate was 85%, the bacterial abundance decreased. The soil inorganic nitrogen N-NH_4_^+^ and N-NO_3_^−^ increased significantly by 11–21%. This significantly improved root growth and plant biomass for the reconstructed soil. The dominating bacterial community was driven by both root exudate components and plant root growth. Especially, the abundance of soil bacteria Actinobacteriota, Proteobacteria, and Chloroflexi was significantly promoted. Consequently, root exudates can be used to efficiently increase the soil fertility and improve the function and vegetation restoration in the soil reconstruction of mines.

## 1 Introduction

Open-pit mining is globally the main coal mining method. Many countries have realized the importance of land reclamation and have successively promulgated laws and regulations with respect to land reclamation of abandoned mining sites. Soil reconstruction is the core of land reclamation, and the quality of soil reconstruction directly determines the land reclamation status (Wang et al., 2021). The original topsoil is the best choice to reconstruct the soil. However, the poor soil development in mining areas due to mining activities causes serious problems of surface soil scarcity (Robson et al., 2018). When topsoil is scarce, suitable soil substitute materials are often screened from the geotechnical layer above the coal seam (Nicolini and Topp, 2005; Wilson-Kokes et al., 2013). Open-pit coal mines in China are mostly located in arid and semi-arid areas. Numerous domestic and foreign research results have shown that the native soil in open-pit mines is deficient in nutrients, and the soil fertility and plant diversity are poor, which is insufficient to realize the functions of soil nutrient cycling and microbial regulation (Wang et al., 2022; Feng, 2019; Hamid et al., 2022). The types of green vegetation available for ecological restoration are limited (Wu et al., 2023; Zhao et al., 2023). Thus, it is necessary to find an amendment for surface reconstruction soil that can balance the requirements of soil microorganism reproduction and the growth needs of locally adapted plants.

Root exudates are the general term for organic compounds released from the roots to the growth medium during the growth and development of plants, which can generally account for more than 10% of the photosynthetic carbon sequestration of plants (Haichar et al., 2016). Root exudates include low molecular weight organic substances, high molecular weight mucilage substances, root cell abscission, and its decomposition products, as well as gases, protons, and nutrient ions (Jiang et al., 2022). They are key factors in regulating the micro-ecological functions of the rhizosphere and also the main medium for communication between plants and soil (Chen et al., 2014; Pausch and Kuzyakov, 2018). They are of great significance for the elemental cycling of soil in the reclamation of mining areas, the absorption of plant nutrients, and the improvement of microbial functions (Ulbrich et al., 2022; Damin et al., 2008). A large number of previous empirical studies have shown that the growth of plants under the influence of root exudates is relatively good (Carla et al., 2020; Viviane et al., 2021). To adapt to the soil environment, plants will adjust the microbial community’s absorption and transformation of nutrient elements by changing the secretion of their root systems (Qu et al., 2020; Sun et al., 2021; Wang et al., 2023; Rahman et al., 2021). Plant root secretion is influenced by biological factors (plant species genotype) and abiotic factors (soil properties, climatic conditions) (Huang et al., 2019; Hu et al., 2021). The components of root exudates secreted by different kinds of plants grown under specific soil conditions are variable (Alberto et al., 2019; Sun et al., 2018). In the rhizosphere soil, bacteria have an absolute preponderant number of rhizosphere microorganisms, which plays an important role in maintaining plant growth and reducing the influence of environmental stress on plants (Jones et al., 2017; Meihong et al., 2019). Plant roots affect the population structure and diversity of rhizosphere bacteria by regulating the type and quantity of root exudates (Cordovez et al., 2024). Rhizosphere bacteria regulate the secretion of plant roots by regulating soil nutrients, thus affecting the growth and development of plants and nutrient absorption (Lyu et al., 2024). The nutrient content, transformation, and utilization efficiency of thin reconstructed soil in mining areas are often low, and the biological adaptability of the reconstructed soil is poor (Ju et al., 2021; Demyan and Smeck, 2022). According to the above, a way to overcome some of these problems may be to use root exudates to recover soil fertility and microbial diversity. It has been shown that low soil microbial biomass can restrict the recovery of soil functioning during the restoration of post-mining lands in semi-arid regions. Thus, especially for mining areas in areas with naturally nutrient-poor soils in arid and semiarid regions, this could be helpful in plant nutrients’ absorption, transformation, and utilization, as an organic modifier and assist microorganism breeding and ecological function recovering in the reconstruction soil land (Lu et al., 2018; Li et al., 2019). The research team selected the typical open-pit mine in China - Zahanoer open-pit mine, Inner Mongolia as the research object. Based on the investigation results, sweet clover (*Melilotus officinalis* L. Pall) was selected as the root exudate extraction body because it is a common reclamation species in mines and has high economic efficiency. Based on the research findings regarding the growth characteristics of common reclamation plants in open-pit mines and in accordance with experimental requirements, it is determined that *Medicago sativa* (*Medicago Sativa Linn*) exhibits significant salt-alkali tolerance and drought resistance. Its genetic mechanism provides a biological foundation for soil improvement in open-pit mines. *Medicago sativa* demonstrates strong adaptability to the reclaimed areas of open-pit mines. As the “king of forage grass, “its cultivation techniques are well-established, and extensive large-scale application experience has been accumulated in arid and semi-arid regions such as Inner Mongolia. Moreover, the molecular mechanisms and field monitoring technologies related to *Medicago sativa* have developed into a relatively comprehensive research system, facilitating experimental design and data collection. Consequently, this study ultimately selects *Medicago sativa*, a pioneer herbaceous reclamation plant, as the experimental subject to investigate the functions of root secretion improvement and microbial regulation. The results can be used to provide basic technical support for ideas and inspiration in the ecological restoration of opencast coal mine areas.

## 2 Materials and methods

### 2.1 Preparation of root exudates

The overburden of the seam in Zhalute County open-pit coal mine, Inner Mongolia, China (120.91507°E, 44.55592°N), was used to reconstruct topsoil and plant herbage. The reconstruction soil was made up of carbonaceous mudstone, mudstone, coal seam floor earth, subsoil, and sandy loam in a weight ratio of 2:1:2:2:2, respectively. The reconstruction soil contained total carbon (TC) of 148 g kg^−1^, total nitrogen (TN) of 1.5 g kg^−1^, bulk density of 1.32 g·cm^−3^, pH of 7.36, and a particle size of 0.5–3 mm. No farmland soil was used in the process.

To obtain root exudates, we cultivated sweet clover (*Melilotus officinalis* L. Pall.) in a sterile environment. The seeds undergo disinfection via a stepwise disinfection method. Specifically, they were first soaked in 75% ethanol for 2 min, followed by immersion in a 3% H_2_O_2_ solution for 15 min, and subsequently rinsed five times with sterile water to thoroughly inactivate any resistant microorganisms present on or within the seeds. This process effectively prevents contamination during subsequent cultivation. Thereafter, the seeds were planted in cups containing a sponge that had been pre-treated by sterilization at 121°Cunder high pressure for 30 min and dried in a 60°C oven for 24 h to eliminate potential microbial growth caused by condensation. The *Hoagland Nutrient Solution* provided nutrition for plant growth (Table 1). When preparing the nutrient solution, it was filtered and sterilized using a 0.22 μm filter membrane, then filled into sterile glass bottles, and stored at 4°C in the dark for no more than 7 days to prevent the introduction and proliferation of environmental microorganisms. We retained 30 sprouted plants in an illuminated incubator and kept them in light for 14 h a day. On the 30th day after seeding emerged, the sweet clover plants were put into a light-proof beaker with 500 mL ddH_2_O to clean them of nutrients. Then, incubation continued for 24 h by using a 0.45 μm water filter membrane to collect the root exudate solution, and through freeze drying, we obtained solid root exudates. Before use, the 0.45 μm filter membrane was subjected to ultraviolet irradiation for 30 min on both sides to ensure thorough sterilization. Subsequently, the tweezers were immersed in 75% ethanol for disinfection and briefly exposed to a flame for sterilization, followed by cooling to room temperature. These steps were taken to prevent the penetration or adhesion of fungal spores that could result from unsterilized materials or contamination during handling. This process was repeated until enough root exudates (the weight of the collected root system was greater than 300 mg) were collected.

**Table 1:** Alpha of diversity indexes.

No.	Ace	Chao	Shannon	Simpson (10^−2^)
C	1,569 ± 43 d	1,576 ± 61 d	5.08 ± 0.05 c	1.31 ± 0.02 d
R1	1816 ± 32 a	1810 ± 55 a	5.51 ± 0.11 b	1.63 ± 0.01 a
R2	1763 ± 72 b	1757 ± 47 b	5.57 ± 0.05 a	1.42 ± 0.01 c
R3	1732 ± 36 b	1732 ± 44 b	5.58 ± 0.07 a	1.52 ± 0.01 b
R4	1,668 ± 19 c	1,685 ± 13 c	5.55 ± 0.07 b	1.41 ± 0.03 c

“Ace” and “Chao” are indicators of species richness, “Shannon” and “simpon” are indicators of species evenness. The lowercase letters “a, b, c, d” in the table denote markers of statistical significance. Differences between data groups labeled with distinct letters are considered statistically significant (typically *p* < 0.05).

The main constituent substances of root exudates were determined by liquid chromatograph-mass spectrometer (LC–MS), including 12 categories and 31 metabolites. The total carbon and nitrogen were determined by a TOC/TN analyzer. The TC was 64.2%, and the TN was 10.2% (Xie et al., 2018; Linlin et al., 2019).

### 2.2 Field experiments

The field experiments were carried out in the waste dump of the Zhahanao’er Open-Pit Coal Mine in Zhalute County, Inner Mongolia, China. The annual average temperature is 6.0°C, and the mean annual rainfall is approximately 379 mm. Every test field block was covered with 20 cm of the reconstructing soil, sized in 1 m × 1 m. On 15 May 2023, *Medicago sativa* (*Medicago Sativa Linn*) was planted in blocks according to 150 kg ha^−1^ seed. Five treatments and three replicates were designed: control (C, water) and four different root exudates’ application concentrations, as 40% mg/ml (R1), 55% mg/ml (R2), 70% mg/ml (R3), 85% mg/ml (R4), respectively, resulting in 15 field blocks in total.

In total, 100 mL of root exudate solution was applied uniformly onto the soil when *Medicago sativa* was at seeding, branching, and flowering periods, respectively. During the entire plant life period, no fertilizer was added, and the plant was watered properly to keep the soil moist at all times. After 5 months, we collected the 0–20 cm layer of soil with plants for analysis. Soil N-NH_4_^+^ and N-NO- 3 were determined by an AA3 analyzer (Technicon, Germany). Soil organic matter (OM) and TN were determined by an element analyzer (Elementar, Germany).

### 2.3 DNA extraction and processing of pyrosequencing data

DNA was extracted from 0.5 g of each individual replicate soil using a *PowerSoil R DNA isolation kit* (Mo Bio Laboratories, Inc., Carlsbad, CA, United States) (Zhang et al., 2022). The hypervariable region V3–V4 of the bacterial 16S rRNA gene was amplified with the primer pair 338F/806R (Xu et al., 2016). The polymerase chain reaction (PCR) amplification method used *TransGen AP221-02: TransStart FastPfu DNA Polymerase* in a 20 μL system.

DNA extraction, PCR amplification, fluorescence quantification, MiSeq library construction, and MiSeq sequencing were used to determine the community composition of soil bacteria. The production of PCR was detected by 2% of lipid gel in lipid sugar gel electrophoresis, and the library was constructed with the *TruSeqTM DNA Sample Prep Kit*. The qualified library was sequenced by the Silva Database (Release138^1^) on an *Illumina MiSeq PE300* platform (Illumina, San Diego, CA, United States) at *Majorbio Bio-Pharm Technology Co., Ltd*. (Shanghai, China).

The Raw Tags were PE Reads joined after splitting through *FLASH* and filtered by *Qiime* (Version 1.7.0) according to quality (Lozupone et al., 2011). Finally, we found the effective tags through length filtering and chimera deletion (Bokulich et al., 2013). Using a 97% identity threshold, the most abundant sequence from each operational taxonomic unit was selected as a representative sequence for that OTU (Li et al., 2021). OTU clustering analysis and species taxonomy analysis were carried out based on the USEARCH7-uparse method.

### 2.4 Statistical analyses

The alpha diversity indices were calculated using *R* (4.2.1; Simpson, Shannon, ACE, and Chao1, Table 1). The beta diversity was performed by principal coordinates analysis (PCoA) and canonical correspondence analysis (CCA) based on *UniFrac* distance to compare the differences between the groups. *R statistics analysis software* and *Origin statistics analysis software* were carried out to calculate standard deviation, LSD, and Tukey’s multiple range test through univariate statistical analysis and multivariate statistical analysis methods and to plot figures. The difference in means at a *p-value of* < 0.05 was considered statistically significant.

## 3 Results

### 3.1 Reconstituted soil fertility and plant growing

**Figure 1:**
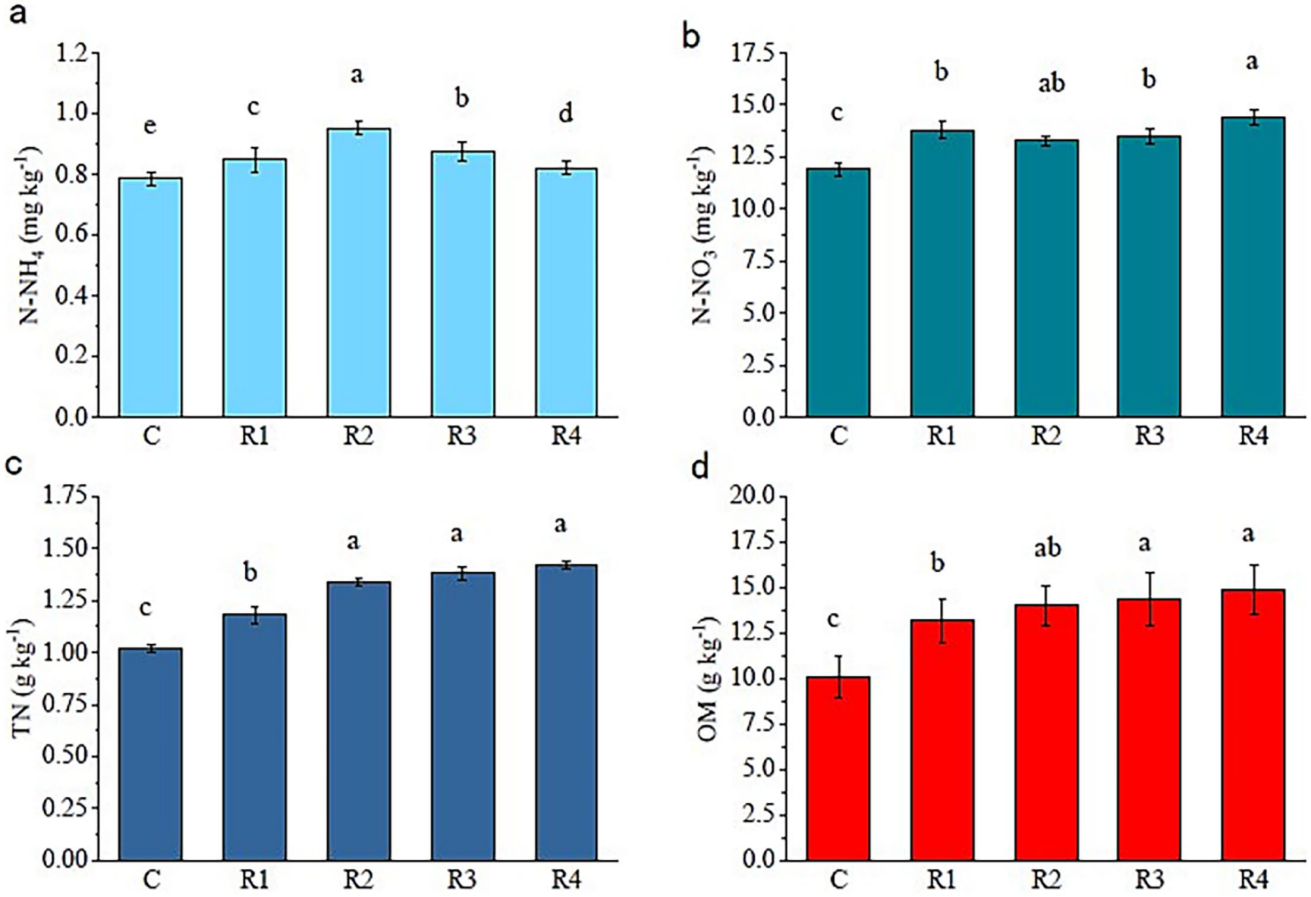
Fertility of soil amended with root exudates. The letters “a, b, c, d” markers of statistical significance. Differences between data groups labeled with distinct letters are considered statistically significant (typically *p* < 0.05).

The concentration of soil TN and OM significantly increased with the increase in the root exudates’ amount (Figure 1). The N-NH_4_^+^ concentrations showed a trend of R2 > R3 > R1 > R4 > C, and the average growth rate of N-NH_4_^+^ was 11.3% (Figure 1a). The N-NO- 3 of R4 was the highest, corresponding to 20.8% higher than the control (Figure 1b). Compared with control, the root exudates’ application increased soil TN from 15.7 to 39.2% (Figure 1c). After using root exudates, reconstruction soil N-NH_4_^+^ and N-NO– 3 increased significantly. There was no significant difference between the N-NO- 3 of R1, R2, and R3. The OM of R4 and R3 was higher than R1 (Figure 1d).

**Figure 2:**
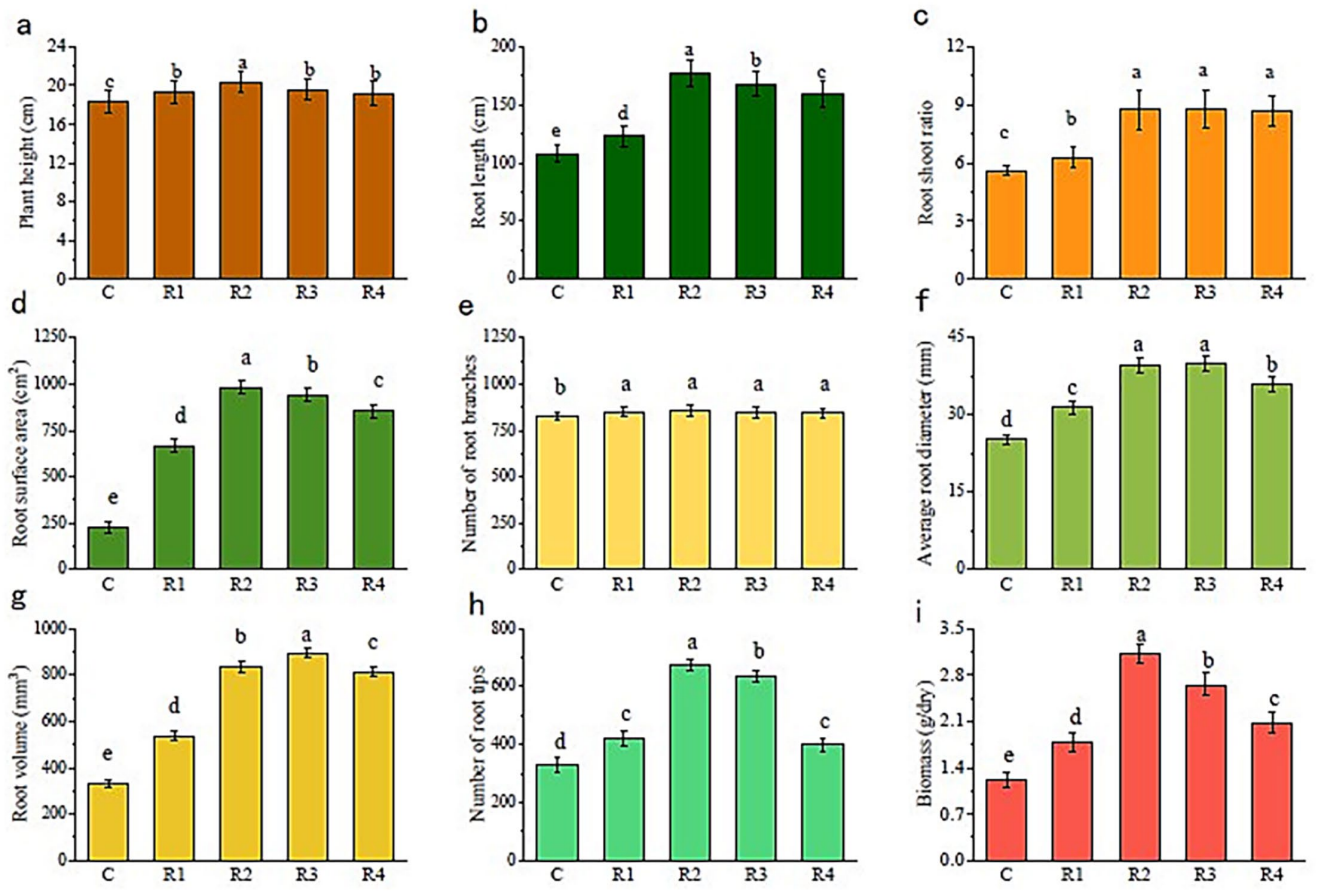
Plant root and biomass growth. The letters “a, b, c, d” markers of statistical significance. Differences between data groups labeled with distinct letters are considered statistically significant (typically *p* < 0.05).

**Figure 3:**
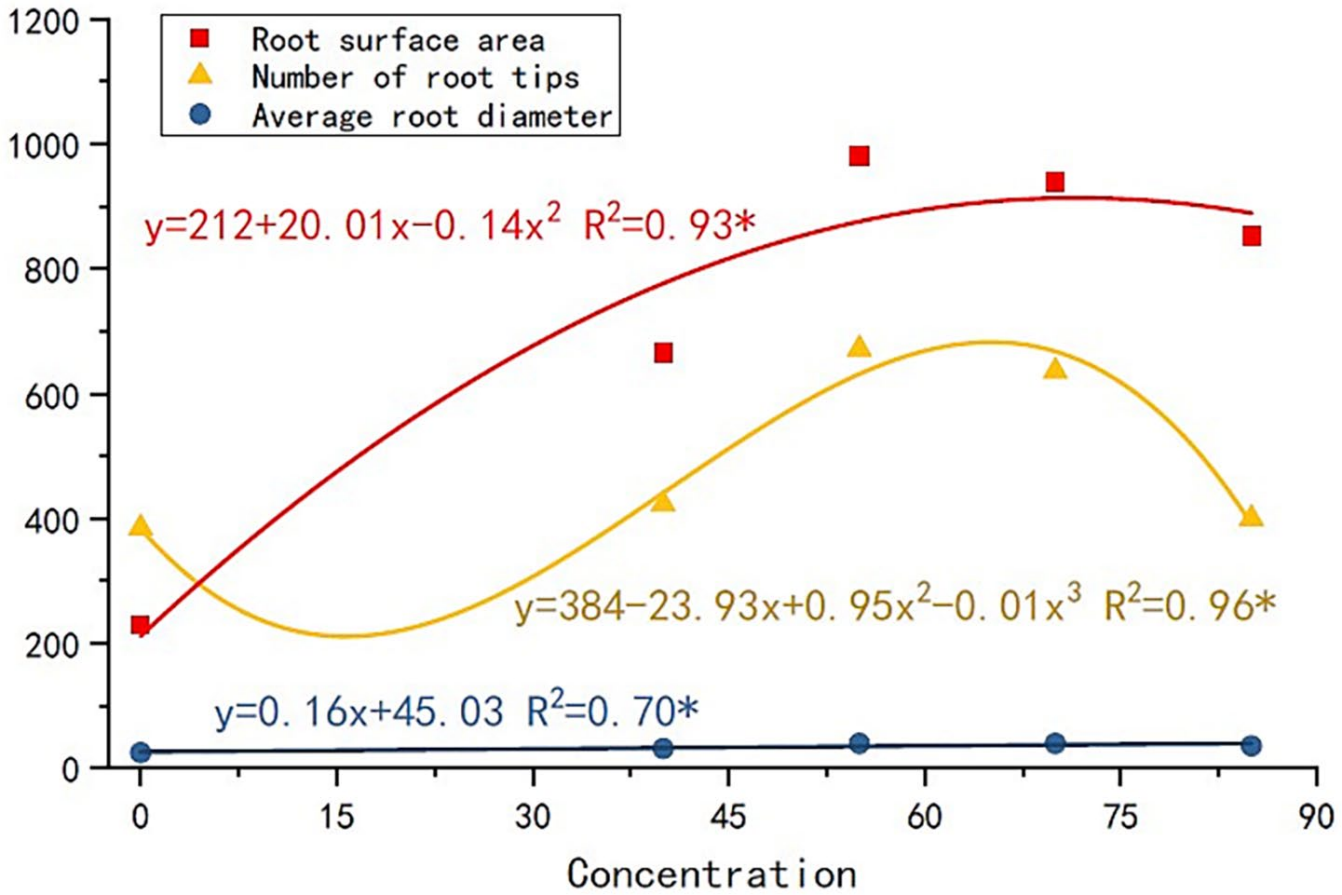
Correlation between the amount of root exudates applied and plant root growth.

The nourishing of root exudates made plant and root growth better than the control (Figure 2). The plant height and root length of R2 were the highest. The plant height of R1, R3, and R4 was similar, while the root length showed a trend of R3 > R4 > R1. The root-shoot ratio was significantly larger for root exudate treatments than for the control. Thus, the application of root exudates helped the plant root to grow a stem. Furthermore, treated plants’ root surface (Figure 2d), volume (Figure 2g), diameter (Figure 2f), number of root tips (Figure 2h), and branches (Figure 2e) were all higher than those of the control (R2 and R3 were always higher than the other). The correlation between the amount of root exudates applied and plant root growth: the root surface area, number of root tips, and average root diameter increased with the increase in the root exudates’ amount (Figure 3). Thus, the root growth resulted in increasing plant biomass (Figure 2i). The biomass and the root length followed the trend R2 > R3 > R4 > R1 > C.

The pH of the treated soil was 7.45, and there was no difference between the different amendments.

### 3.2 Soil bacteria community composition

**Figure 4:**
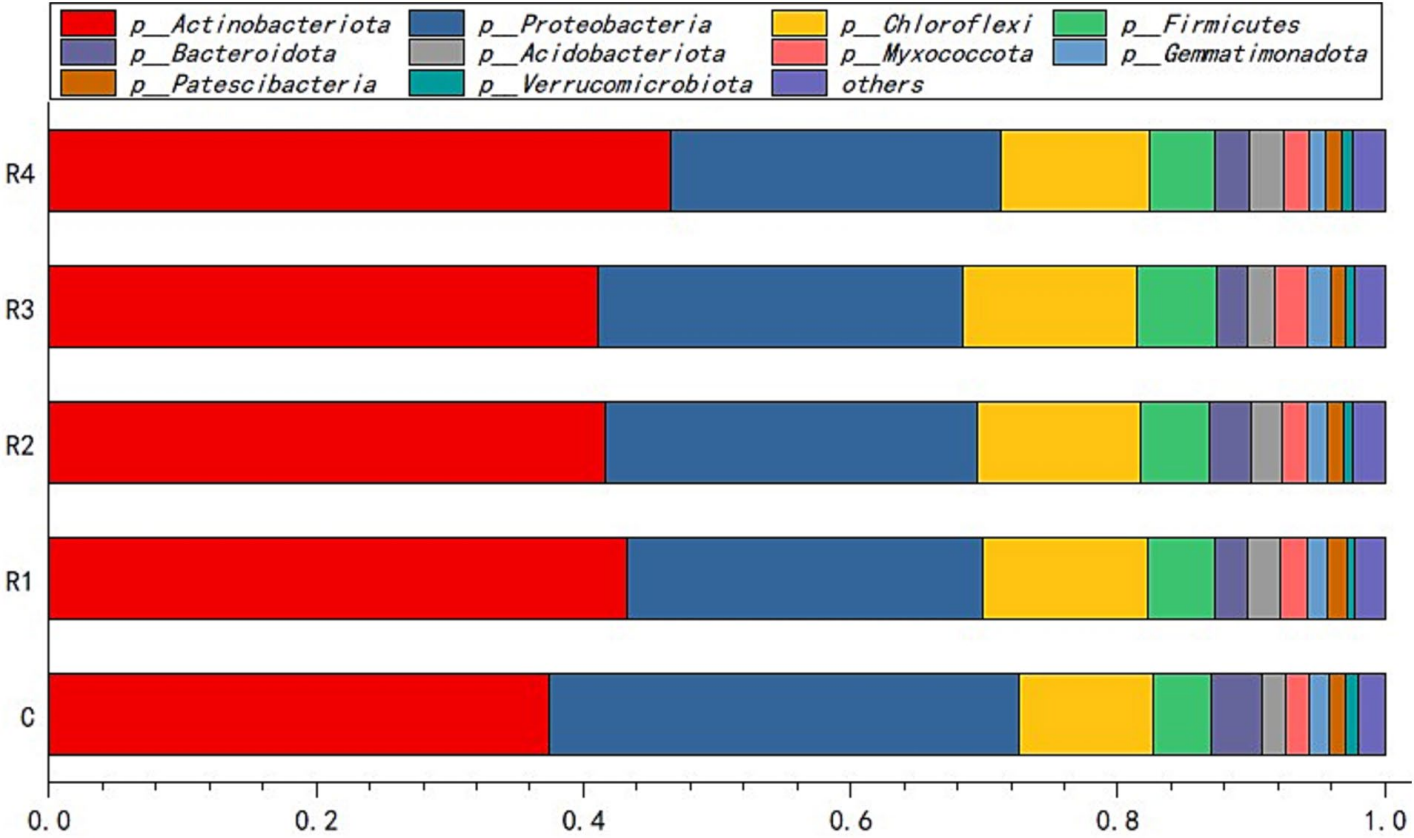
Soil bacteria relative abundance of the phylum. “p_” is the abbreviation of “Phylum”.

*Actinobacteriota* and *Proteobacteria* were the dominant bacterial species in the amended soil. The sum of their relative abundance in the soil samples of each treatment was more than 50% (Figure 4). *Chloroflexi* and *Firmicutes* were the third and fourth largest relative type, respectively. The other bacteria types did not exceed 15%.

**Figure 5:**
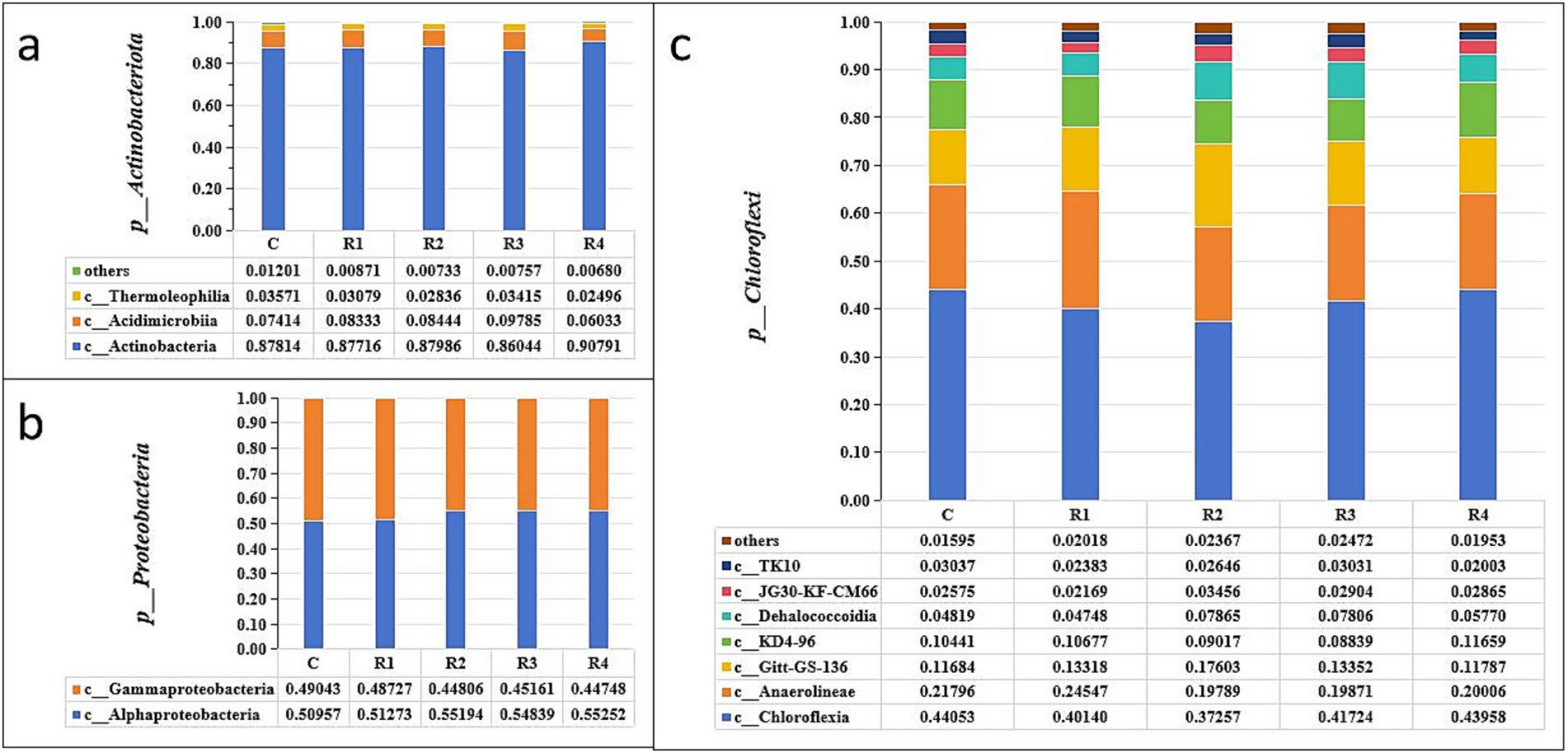
Relative abundance of soil Actinobacteriota **(a)**, Proteobacteria **(b)**, and Chloroflexi **(c)**. “p_” is the abbreviation of “Phylum,” “c_” is the abbreviation of “Class”.

**Figure 6:**
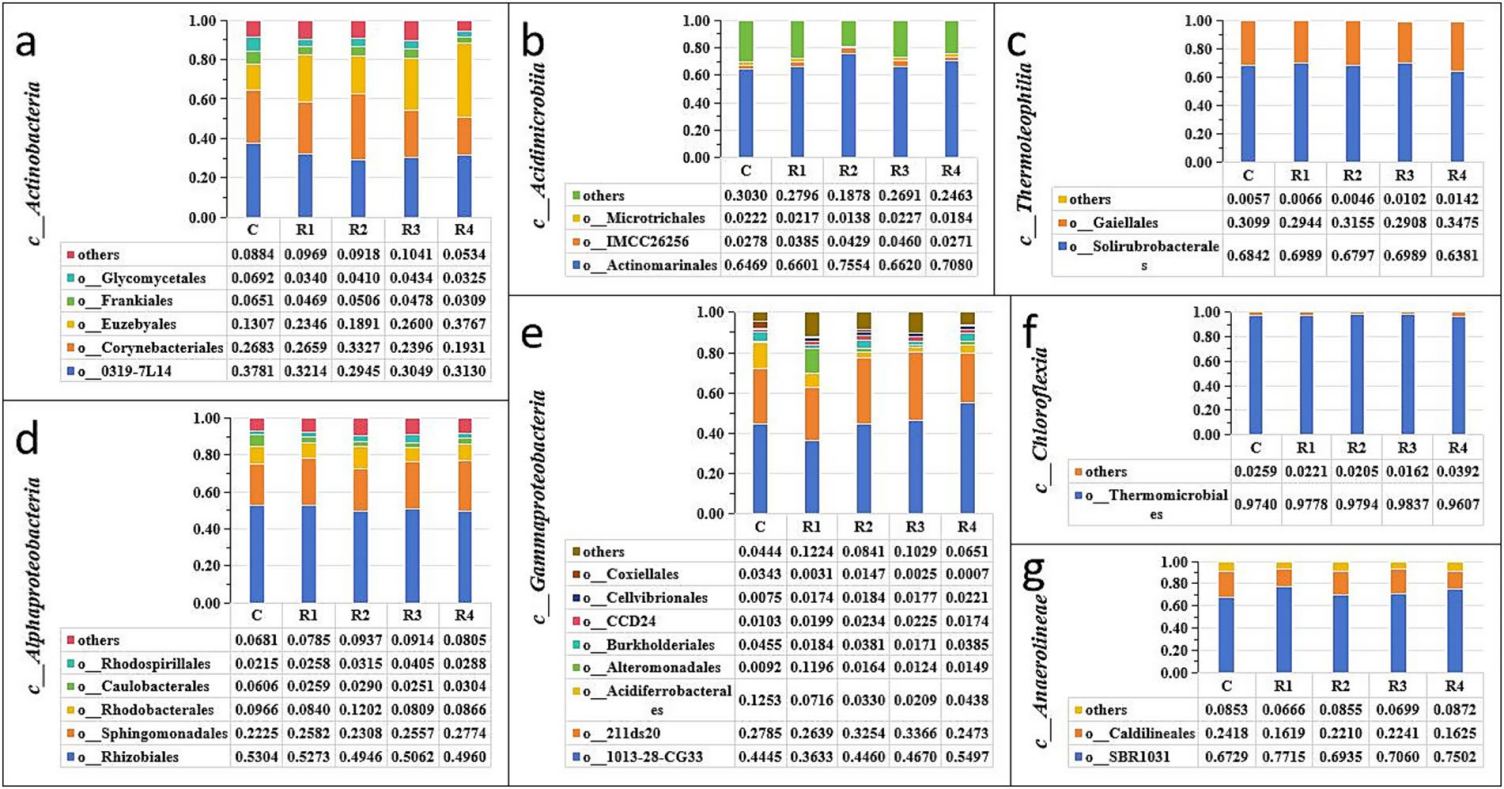
Relative abundance of soil Actinobacteria **(a)**, Acidimicrobiia **(b)**, Thermoleophilia **(c)**, Alphaproteobacteria **(d)**, Gammaproteobacteria **(e)**, Chloroflexia **(f)**, and Anaerolineae **(g)**. “c_” is the abbreviation of “Class” and “o_” is the abbreviation of “Order”.

After root exudates’ application, the *Actinobacteriota* increased significantly, especially in R4, which was 15% higher than in the control (Figure 4). It can be seen that the root exudates significantly promoted the relative abundance of the *Actinobacteria* class (Figure 5a) and the *Euzebyales* order (Figure 6a). The classes *Acidimicrobiia* and *Thermoleophilia* were the other main bacterial classes (Figures 6b,c). The *Actinomarinales* order of *Acidimicrobiia* class in the R2 treatment was higher than others. The *IMCC26256* and *Microtrichales* orders of the *Acidimicrobiia* class, as well as the *Solirubrobacterales* and *Gaiellales* orders of the *Thermoleophilia* class, were not different among the different root exudates’ amount treatments.

In contrast, the relative abundance of *Proteobacteria* was the highest under the control treatment, and lowest under the R4 treatment (Figure 4). The main *Proteobacteria* were the *Alphaproteobacteria* and *Gammaproteobacteria* (Figures 5b, 6e) (Figure 5b). There was no difference among treatments for the *Alphaproteobacteria* class (Figure 6d). The exudates amendment of R1 reduced the *1,013-28-CG33* order but increased the *Alteromonadales* order. After root exudates were added, the number of orders under *Gammaproteobacteria* increased, and the increased bacterial orders were *Alteromonadales*, *Burkholderiales*, *CCD24,* and *Cellvibrionales*. The relative abundance significantly decreased for *Alteromonadales* with the addition of root exudates.

The *Chloroflexi* relative abundance after root exudates’ amendment increased slightly compared with the control (Figure 4). *Chloroflexia* and *Anaerolineae* were the main bacterial classes of the *Chloroflexi* phylum (Figure 5c). Further analysis for the orders of *Thermomicrobiales*, *SBR1031,* and *Caldilineales* showed the different amendment did not alter the relative abundance and community (Figures 6f,g). This indicates that root exudates helped the bacterial species *Chloroflexia* growth.

### 3.3 Bacterial diversity and abundance

**Figure 7:**
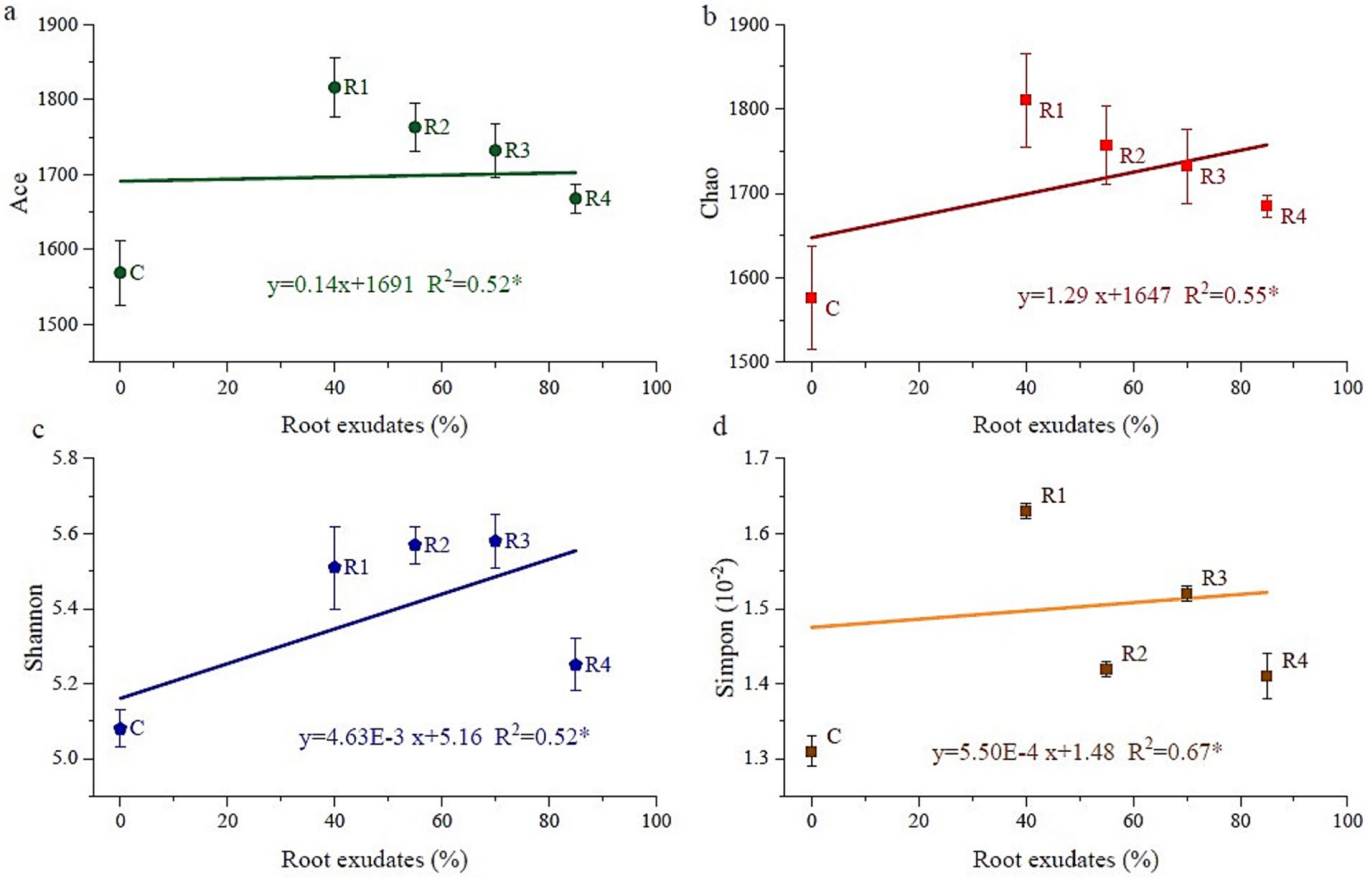
Correlation between the amount of applied root exudates and alpha bacteria diversity. “Ace” and “Chao” are indicators of species richness, “Shannon” and “simpon” are indicators of species evenness.

The different root exudates’ amendments and control all had a coverage rate of over 99% of the bacterial community in the tests. According to the alpha diversity results, root exudates’ amendment increased the alpha diversity indices (Figure 7). The Ace and Chao index of R1 was the highest, and then R2, R3, and R4. R2 and R3 had the highest Shannon index, followed by R1 and R4. However, the Simpson index showed a trend of C < R4, R2 < R3 < R1. According to the correlation analyses (Figure 7), the alpha diversity was linearly correlated with root exudate concentration. To summarize the alpha diversity results, root exudates’ amendment tends to increase the homogeneity of developed soil bacteria species. It promotes local existing soil bacterial growth but does not stimulate new bacterial species. Thus, the number of soil bacteria increases rather than enhancing the species number.

**Figure 8:**
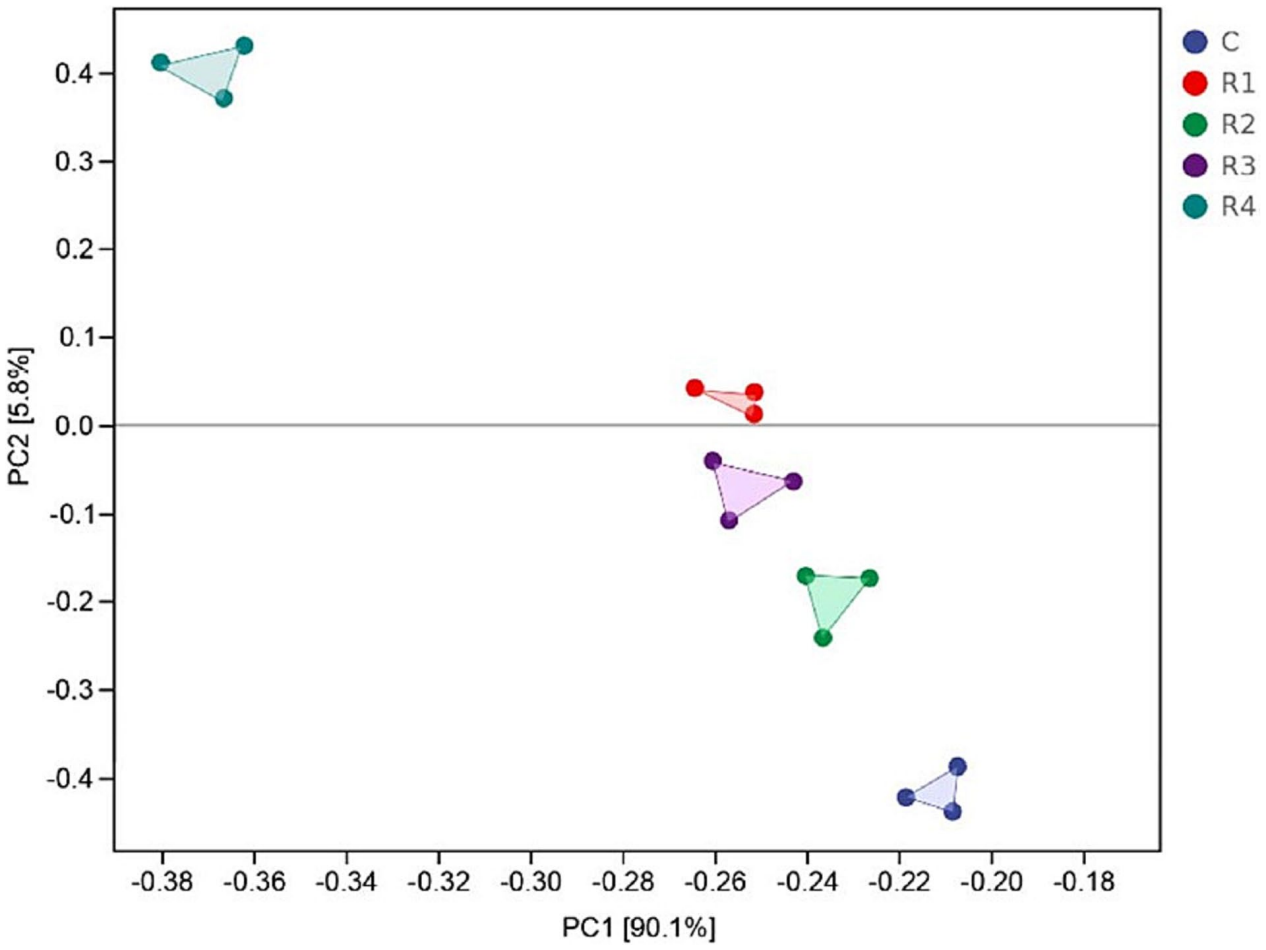
PCoA analysis of amended soil bacterial communities.

The PCoA analysis evaluated the between-habitat diversity of amended soil bacteria (Figure 8). The first two principal components, PC1 and PC2, together explain 95.9% of the total variation in the bacterial community. The treatments by root exudates were significantly divided. R1, R2, and R3 gathered more closely than R4. The reason for this phenomenon might be that the content of root exudates has differential effects on bacterial diversity. When the concentration of root exudates ranges from 40 to 70%, bacterial diversity is at its highest. However, when the concentration of root exudates is 85%, the addition of root exudates leads to an imbalance in the utilization of soil nutrients by bacteria, thereby reducing bacterial diversity and causing a decrease in bacterial abundance.

### 3.4 Relationships between amended soil bacterial communities

**Figure 9:**
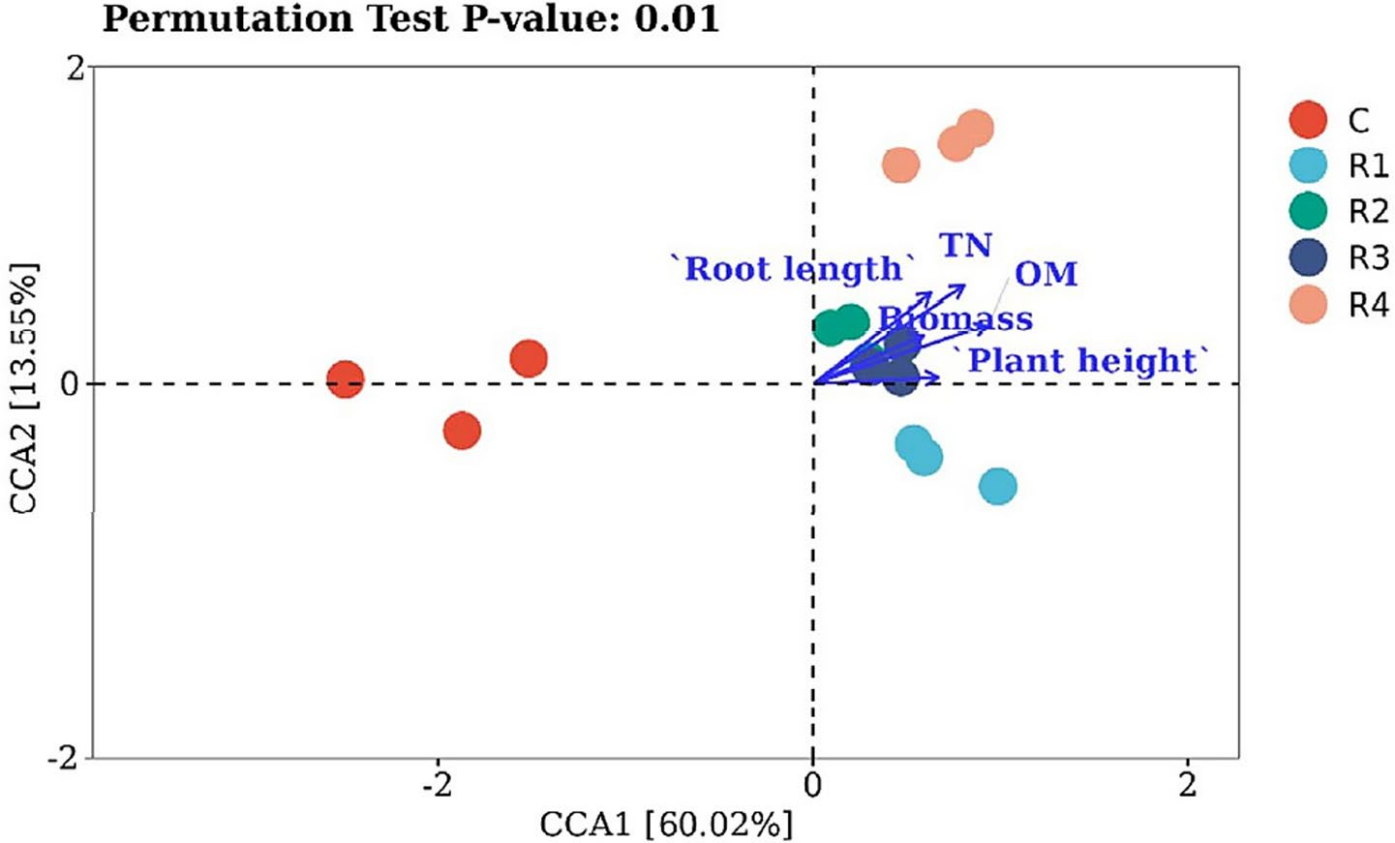
CCA analysis of amended soil bacterial communities.

Canonical correspondence analysis was used to reveal what environmental factors changed the bacterial communities in the amended soil (Figure 9). The five treatments resulted in different bacterial distributions. The control was far away from amendments. The root length and plant height were positively associated with amended soil TN and OM. The shifts in bacteria OTUs were correlated with environmental variables. The root exudates changed the amended soil bacteria community by increasing plant root growth and soil fertility from increased TN and OM. R2 and R3 were significantly different from R1 and R4 in terms of biomass and fertility, in which R1 has the strongest promoting effect and R4 the weakest. The root exudates, thus, accelerate the transfer of soil nutrition to soil microorganisms.

## 4 Discussion

A main ecological problem from open coal mine use is restoring the soil surface environment. This is due to the shortage of topsoil and low soil fertility in many arid and semi-arid regions. Soil improvement is a critical component of ecological restoration in open-pit mining areas (Cantó et al., 2020; Malik and Jawad, 2022). Traditional fertilizers and root exudates, as two primary soil improvement methods, exhibit significant differences in their mechanisms and effects. Traditional fertilizers include chemical and organic fertilizers. The mechanism of chemical fertilizer is to improve soil fertility by directly supplementing nitrogen, phosphorus, potassium, and other nutrients in the soil (Tong et al., 2024). Studies have shown that chemical fertilizers can significantly increase the content of available nutrients in soil; for example, nitrogen fertilizers can raise soil alkali-hydrolyzable nitrogen content by 30–50% (Dakora and Phillips, 2002; Badri and Vivanco, 2009). However, the long-term application of chemical fertilizers may lead to adverse effects, such as soil acidification, compaction, and reduced microbial diversity (Wan et al., 2018).

The mechanism of action of organic fertilizer is to convert organic matter into humus through microbial decomposition, thereby improving soil structure and enhancing its water-holding and nutrient retention capacity (Wen et al., 2020). Research shows that the application of organic fertilizer can significantly increase soil organic matter content. For instance, applying cow manure or biogas slurry can increase the soil organic matter content by more than 30% (Hammad et al., 2020). Moreover, organic fertilizer can promote the formation of soil aggregates, improve soil aeration, and enhance the water–air ratio (Wang et al., 2024). However, the improvement effect of organic fertilizer is relatively slow, and its contribution to enhancing soil microbial diversity is limited (Tittonell et al., 2008). Root exudates indirectly improve soil structure and quality by activating insoluble nutrients and promoting microbial activity. Organic acids (e.g., oxalic acid and citric acid) in root exudates facilitate the dissolution of insoluble minerals through acidolysis and chelation (Bais et al., 2006). For instance, root exudates from cypress can increase soil available phosphorus content by 20–30%. Additionally, root exudates provide abundant microbial nutrients and carbon sources, enhancing rhizosphere microbial metabolic activity and accelerating nutrient mineralization and transformation (Li et al., 2012; Deru et al., 2023). Polysaccharides in root exudates also promote the formation of soil aggregates, thereby improving soil structure (Zeng et al., 2008; Yang et al., 2024). Through literature review and data comparison (Hao et al., 2022; Nannipieri et al., 2008; Shen et al., 2019), the soil improvement effect indicators of traditional fertilization and applied root secretion were quantitatively analyzed and compared, as shown in Table 2.

**Table 2:** Comparison of the effect data of different methods.

Index	Chemical fertilizer	Organic fertilizer	Root exudates
Alkali-hydrolyzed nitrogen	Increased by 30–50%	Increased by more than 30%	Increased by 10–20%
Available phosphorus	Increased by 20–40%	Increased by 20–40%	Increased by 20–30%
Available potassium	Increased by 25–35%	Increased by 25–35%	Increased by 15–25%
Microbial diversity	May reduce	Shannon index: Increased by 5–10%; Number of OTUs: Increased by 10–15%	Shannon index: Increased by 15–25%; Number of OTUs: Adding 20–30%
Structure improvement	No significant improvement	Aggregate stability: Increased by 10–20%; Porosity: No significant change; Proportion of water-stable aggregates: Increased by 5–10%	Aggregate stability: Increased by 30–40%; Porosity: Increased by 15–25%; Proportion of water-stable aggregates: Increased by 20–30%

In summary, although traditional fertilization has relatively better immediate effects on improving soil nutrients, considering the flexibility and sustainability of the improvement effect, the improved root secretion method is more suitable for open-pit soil with poor nutrients, high soil strength, and low microbial activity (Table 2) (Deonalli et al., 2017; Zhao et al., 2025; Keiluweit et al., 2015). In this study, root exudates were used as soil amendments to improve the soil content of C and N in an abandoned open-pit coal mine area. Soil OM is the limiting factor to the growth of both plants and microbes (Lal, 2020). The root exudates contain vitamins, peptides, organic acids, lipids, carbohydrates, and other important plant organic nutrient substances. These substances promote root growth, absorb more water from the soil, and support the whole plant growing stage (Han et al., 2018). Furthermore, secondary metabolites in root exudates can help plants adapt to poor fertility and the biotic stress of impoverished soils (Vives-Peris et al., 2020). The results of this study also showed that the higher the amount of root exudates, the higher the soil fertility and root-shoot ratio.

Microorganisms play an important role in the nutrient transformation between amended soil and plants. However, the microorganism density is low with small diversity, especially in the mine ecosystem (Zhou et al., 2018). Previous studies have shown that root exudates can stimulate the growth of microorganisms and improve soil bacterial abundance (Zhou et al., 2012). When root exudates are sufficient, they can provide more energy for microbial metabolism and improve the abundance of soil bacteria, which is consistent with the conclusion of this study. On the one hand, the improved fertility of amended soil provides a substrate that is easily decomposed by microorganisms (Clocchiatti et al., 2021). The soil bacteria density increased with the continuous supply of nutrients (Figure 7). This was shown by the positive correlation between soil bacterial community distribution and soil fertility from OM and TN (Figure 9). Furthermore, increased plant growth by adding root exudates improved the ecological function of amended soil (Parray et al., 2016). Plant growth promotes rhizobacteria interactions with plants, such as element fixation and solubilization of N and P (Rosier et al., 2018), as well as improving plant drought resistance and the antiviral ability in the rhizosphere (Morales et al., 2022). Thus, root exudates improve soil bacteria by promoting plant growth, especially root growth (Ge et al., 2017).

In our experiments, the local dominating bacteria community was driven by root exudates and plant growth, as also shown in previous studies (Freedman et al., 2015; Lanoue et al., 2010; Maurer et al., 2021). The bacteria were sensitive to nutrients and organic matter in poor soils (Badri and Vivanco, 2009). The results of this study indicate that the bacterial diversity was highest for a root exudate concentration of 40–70%. If the added root exudates cause an imbalance between C and N in terms of bacterial use, the diversity of bacteria could be reduced (Jiang et al., 2021). Consequently, bacterial abundance decreased for a root exudate concentration of 85%.

A high concentration of root exudates was most obvious in promoting *Actinobacteriota*, *Proteobacteria,* and *Chloroflexi*. *Actinobacteriota* are an important bacterium in soil. Its slender and complex mycelia contribute to the formation of soil aggregates and the accumulation of organic carbon (Xie et al., 2022), which is of great significance to the improvement of basic soil fertility (Hernández-Álvarez et al., 2022). *Proteobacteria* are the most widely distributed and complex functional flora in farmland and natural soil. *Proteobacteria* help the fixation and transformation, especially mineralization, in the soil N cycle (Li et al., 2022). *Alphaproteobacteria* and *Gammaproteobacteria* are closely related to plant N-uptake and utilization from soil (Bugg et al., 2011). *Chloroflexi* is a facultative anaerobic bacteria that can generate energy through photosynthesis (Wang et al., 2019). It does not produce oxygen or fix nitrogen in photosynthesis, but it is helpful for the circulation of soil nutrients (Speirs et al., 2019). *Firmicutes* can produce spores with strong resistance to drought and extreme environments. All abundant bacteria in our experiments can adapt well to harsh soil conditions (Hernández-Álvarez et al., 2022). They, as well, can improve the efficiency of amended soil to reconstruct and enhance plant growth.

Activating soil nutrients through root exudates and promoting the absorption of soil nutrients by crops is of great significance for improving the productivity of poor soil. The soil in open-pit mining areas is nutrient-poor, making it essential to select locally adapted reclamation plants that are most suitable for growth in the planting system. Additionally, the impact of root exudates from these reclamation plants on soil properties and plant growth should be considered. Direct or indirect effects of root secretion from reclaimed plant species can affect the growth and development of their own or neighboring crops, improving the properties of soil under nutrient stress, and enhancing the effect of plant reclamation. The findings regarding the interaction between plant root exudates and rhizosphere microorganisms in this study hold significant theoretical and practical value for elucidating soil ecosystem functions and guiding vegetation restoration and ecological management in mined areas. In the future, the appropriate ecological planting model and adaptation mechanism of plants mediated by plant root exudates can be systematically studied to guide the ecological planting work in mines.

## 5 Conclusion

Applying root exudates in impoverished soil ecosystems of open-pit coal mines could increase soil fertility and improve the plant nutrient status. In the amended soil, the root exudates can simultaneously promote plant growth and bacterial proliferation. The growing plant roots will gradually increase bacterial abundance and diversity. The local dominant bacterial species of soil nutrient cycling increased and became more abundant. In a certain range, increasing the amount of root exudates can significantly increase the bacterial abundance.

This study demonstrates the practical application feasibility of root exudate-mediated soil amendment technology in the ecological restoration of open-pit coal mines. However, prior to large-scale implementation, region-specific analyses integrating pedological characteristics, reclamation objectives, and monitoring technologies must be conducted. A three-phase implementation strategy should be rigorously followed. In the initial restoration stage, concentrated root exudate solutions should be sprayed preferentially to activate the local microbial community and create a micro-environment for subsequent plant colonization. In the mid-term collaborative stage, synchronized with the sowing of stress-tolerant plants, continuous input of low-concentration exudates from the drip irrigation system should be adopted to promote root development and colonization of microorganisms. In the later maintenance stage, soil microbial diversity should be regularly monitored, and the amount of exudate application should be adjusted according to the abundance of dominant bacteria to avoid excessive stimulation that leads to community imbalance.

## Data Availability

The original contributions presented in the study are publicly available. This data can be found here: https://data.mendeley.com/datasets/jm2gcrf66p/1.

## Footnotes

^1^ http://www.arb-silva.de
